# Endovascular treatment with in-vitro fenestration and sac filling technique for ruptured thoracoabdominal aortic aneurysm with Behcet’s disease

**DOI:** 10.1186/s13019-020-01252-6

**Published:** 2020-08-17

**Authors:** Shibo Xia, Chao Song, Lei Zhang, Wenping Hu, Haiyan Li, Yu Shen, Qingsheng Lu

**Affiliations:** Department of Vascular Surgery, Changhai Hospital, The Second (Navy) Military Medical University, CPLA, 168 Changhai Road, Shanghai, 200433 China

**Keywords:** Behcet’s disease, Ruptured Thoracoabdominal aortic aneurysm, In-vitro fenestration technique, Sac filling technique, Endovascular treatment

## Abstract

**Purpose:**

We provided an endovascular strategy of treating ruptured aortic aneurysm with Behcet’s Disease.

**Case report:**

A 25-year-old man was diagnosed ruptured thoracoabdominal aortic aneurysm with Behcet’s Disease according to his eye damage history, high level of ESR and C-reactive protein and the imaging result. We used in-vitro fenestration of the stent-graft combined with in-stent technique to occlude the ruptured aortic aneurysm and preserve the blood supply from the aorta for visceral arteries in emergency. Sac filling technique was used to treat the endoleak to quickly prevent bleeding. The patient kept post-operative immunotherapy for 1 year.

**Conclusion:**

The patient had a good prognosis in the reduction of the cavity of aortic aneurysm to the normal size without any complications in a year follow-up.

## Introduction

Behcet’s disease (BD) is a systemic immune disease, and vascular involvement is a common complication, which affects up to 40% of BD patients. Among all arterial complications, aortic aneurysm is the most common and severe one [1, 2]. Because of high risk of postoperative complications with open surgery, endovascular treatment is considered for aortic aneurysm. However, endovascular treatment for thoracoabdominal aortic aneurysm (TAAA) is difficult since the strategies of preserving the visceral artery involved by aortic aneurysm and preventing endoleak are necessary.

We report this case of ruptured TAAA with BD that treated with in-vitro fenestration to preserve the visceral artery involved by aortic aneurysm and sac filling technique to prevent endoleak.

## Case report

The patient was a 25-year-old male who had sustained sudden severe pain in the left lower back and radiated to the scapular area for 4 h, accompanied by breathing difficulties with shock. The CT scan confirmed a ruptured TAAA (Crawford type IV). Bilateral lens exchange surgery was operated 1 year ago. According to features like age, the past ocular damage, C-reactive protein level of 71.5 mg/L and calcitonin level of 0.414 ng/L, TAAA rupture caused by BD was highly suspected.

**Fig. 1:**
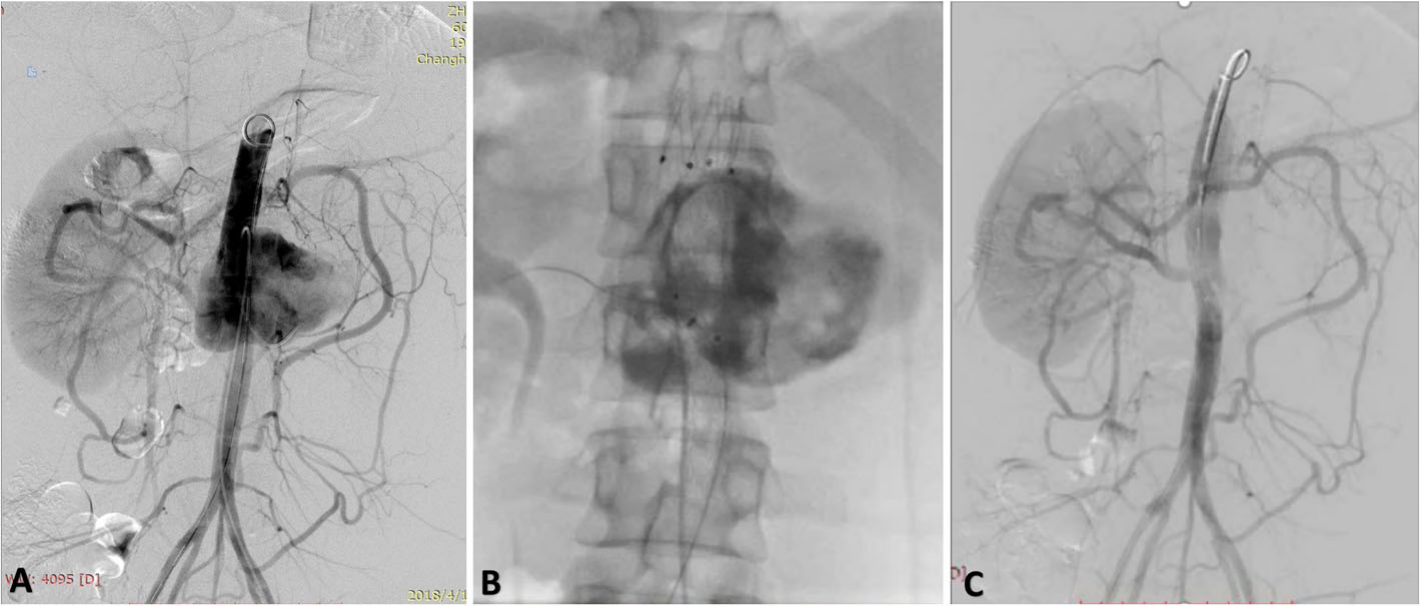
**a**: Preoperative angiography of endovascular treatment. **b**: After fibrin sealant injected, the contrast agent was static in the sac. **c**: Postoperative angiography showed no endoleak

Emergent arteriography demonstrated a huge ruptured TAAA with the maximal diameter of 102 mm. The proximal end of the aneurysm was parallel to the top of the celiac artery. The distal end was 20 mm below the opening of the right renal artery, while was proximal to the inferior mesenteric artery. The ruptured location of TAAA was near the opening of the right renal artery. The celiac artery, left renal artery and superior mesenteric artery were occluded, while the collateral circulation was from the inferior mesenteric artery (Fig. 1a). The maximal diameter of proximal aorta above the celiac artery was 21.2 mm, the diameter of distal aorta below the right renal artery was 15.2 mm and that of the proximal right renal artery was 6 mm measured by arteriography and CT.

Because of the different upper and lower diameters of the abdominal aorta in the lesion, we chose a bifurcated stent-graft (ENDURANT, Medtronic, USA, 23–16-145 mm). The main body of the abdominal aortic stent-graft was deployed on the back table in the operating room. Fenestration (6*6 mm) was then performed at 5 cm from the proximal top of the stent graft, and in the 9 o’clock direction with the radioactive “8” mark at the edge of the hole. The short bifurcated component of the stent-graft was occluded. The in-vitro fenestrated stent-graft was re-sent into the delivery system. The stent-graft was delivered from the right femoral artery to the aorta over the super-stiff guidewire, then deployed in the right position of the fenestration facing to the opening of the right renal artery. The arteriography demonstrated that the right renal artery was patent from the fenestration, but still with lots of endoleak. Then a covered stent (Viabahn, GORE, USA, 6*20 mm) was put into the right renal artery via the fenestration.

A 5F vertebral catheter was preloaded into the aortic aneurysm sac for filling treatment from the left femoral artery before the aortic stent-graft was deployed. A Coda balloon (Cook Medical, Bloomington, Ind) was used to block proximal blood flow. Then, 30 ml fibrin sealant (Shanghai RAAS Blood Products Co, Ltd., Shanghai, China), including 15 ml fibrinogen (90 mg/ mL) and 15 ml thrombin (500 IU/mL) solutions, was injected into the sac of aneurysm. (Fig. 1b) After the balloon was withdrawn, the arteriography was performed that the proximal end of the stent-graft was above the diaphragm and the distal end of the stent-graft was above the inferior mesenteric artery. The length of the proximal and distal landing zone was more than 4 cm. The right renal artery was patent without any endoleak, and the collateral circulation of the left renal artery and superior mesenteric artery was static from the patent inferior mesenteric artery. (Fig. 1c) the patient was transferred to ICU after the surgery.

**Fig. 2:**
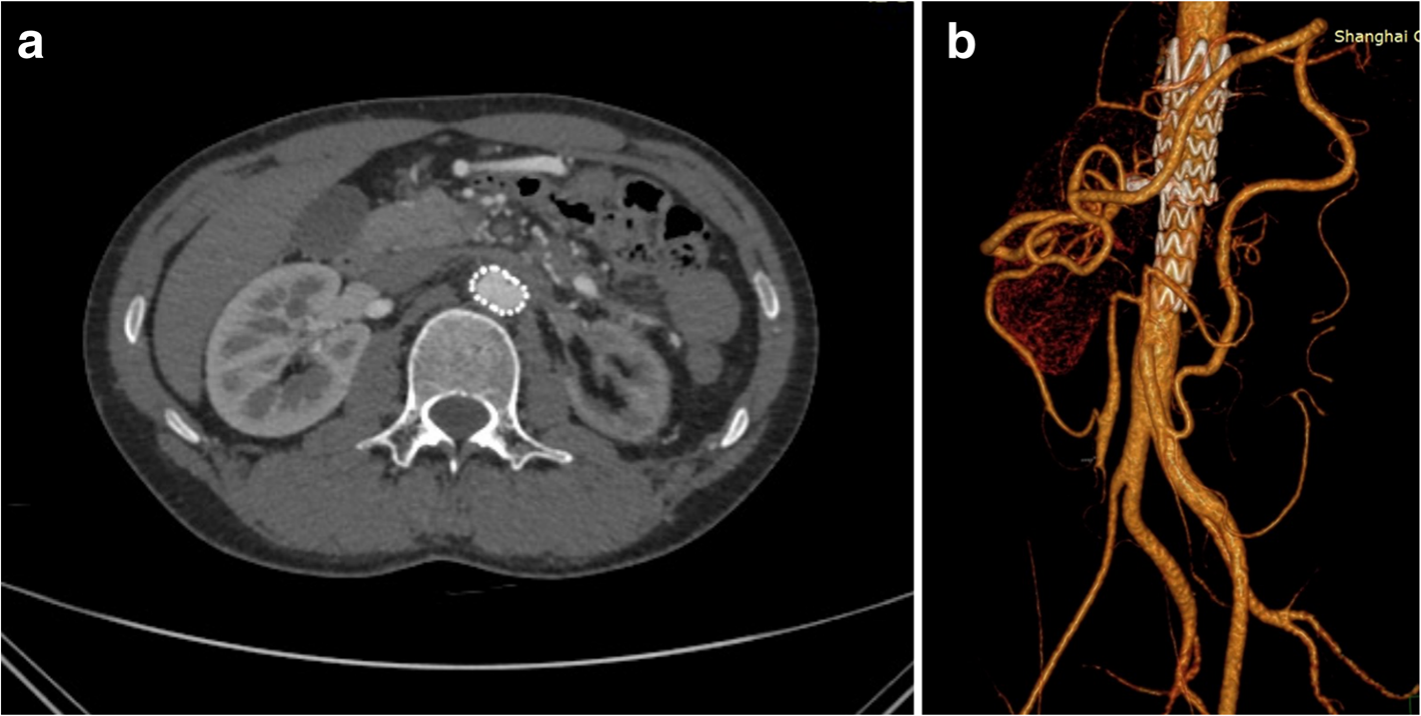
One year postoperative follow-up of the patient showed complete aneurysm reduction and absorption of retroperitoneal hematoma

After the endovascular treatment, the patient had no postoperative complications, such as renal insufficiency, ischemic intestinal symptoms and paraplegia. The hemodynamic function was kept stable. Tetracycline and dexamethasone were used for BD pulse therapy for 3 days. The patient kept taking prednisone for 1 year. The CTA follow-up 1 year after the surgery showed that the diameter of the aortic aneurysm was reduced even to the normal diameter of the artery and retroperitoneal hematoma was absorbed without any endoleak. The right renal artery and the collateral circulation from the inferior mesenteric artery were patent (Fig. 2). The ESR and CRP values were within the normal range.

## Discussion

For the ruptured aortic aneurysm of BD, it is important to diagnose the etiology in emergency for the selection of treatment and for the prognosis. Although there was not enough immunological evidence, the patient was highly suspected of immunologic etiology based on the young age, eye damage history and the imaging result which showed a different morphology from degenerative aortic aneurysm [3]. The preoperative evidence of the high level of ESR and C-reactive protein was further confirmed that the etiology of the ruptured TAAA was related to BD. Furthermore, the etiology of TAAA with BD was also confirmed by the prognosis after endovascular treatment and immunotherapy.

Since Dr. Vasseur [4] first reported the use of bifurcated stent-graft to treat BD with abdominal aortic aneurysms in 1998, more studies have shown that endovascular treatment of ruptured aortic aneurysms caused by BD was practical [5]. However, the details of endovascular treatment of BD’s aortic aneurysm were different from that of degenerative aneurysm.

Because of the inflammation of the proximal and distal neck of the aortic aneurysm, the landing zone was at least 40 mm, which was 15 mm longer than that in the general treatment for aortic aneurysm, to prevent the rupture of the neck of the aortic aneurysm at the landing zone. In order to exert less stimulation on aorta, we selected the stent-graft of the proximal and distal oversize less than 10% which was ordinary 15–20% of treatment for degenerative aortic aneurysm.

The in-vitro fenestration of the stent-graft combined with in-stent technique can effectively occlude the ruptured TAAA and preserve the blood supply from the aorta for visceral arteries. Because the collateral circulation of the left renal artery and superior mesenteric artery was from the inferior mesenteric artery for this patient, it was more convenient to apply this technique just to preserve right renal artery. Fibrin Sealant (FS) is a human plasma extract, which is composed of fibrinogen and thrombin. Our center had the experience of treating AAA with FS and showed good clinical outcomes in preventing postoperative endoleak of AAA. Our research demonstrated that FS sac filling [6] technique can effectively treat any type of the endoleak for FEVAR, especially for ruptured EVAR, to quickly prevent bleeding.

In this case, the prognosis of the patient was better than FEVAR for degenerative aortic aneurysm, with sac reduced to the normal size without any complications in a year follow-up. The reason is that in-vitro fenestration combined with sac filling technique effectively excluded the ruptured TAAA and completely prevented the endoleak. In addition, postoperative long-term immunotherapy kept CRP and ESR at normal levels, which inhibited fibrosis and the inflammation of vascular intima.

## Conclusion

Above all, endovascular treatment combined with postoperative immunotherapy has an excellent prognosis on the ruptured aortic aneurysm with BD. The case report confirms the effect of this method.

## Data Availability

As this is a case report, there is no dataset available.

## Abbreviations

BD: Behcet’s Disease
TAAA: Thoracoabdominal Aortic Aneurysm
CT: Computerized tomography
CTA: Computerized tomography angiography
ESR: Erythrocyte sedimentation rate
CRP: C-reactive protein
EVAR: Endovascular aortic repair
FEVAR: Fenestrated endovascular aortic repair
